# Effects of different microfracture drilling parameters on bone quality: a finite element analysis

**DOI:** 10.3389/fbioe.2024.1515136

**Published:** 2025-01-08

**Authors:** Jiayi Luo, Zihao Zou, Qiang Zou, Siwei Luo, Jialin He, Chuan Ye

**Affiliations:** ^1^ Department of Orthopaedics, The Affiliated Hospital of Guizhou Medical University, Guiyang, China; ^2^ Center for Tissue Engineering and Stem Cell Research, Guizhou Medical University, Guiyang, China

**Keywords:** microfracture, finite element analysis, osteoarthritis of the knee (KOA), drilling, kirschner wire

## Abstract

**Background:**

Microfracture drilling is a surgical technique that involves creating multiple perforations in areas of cartilage defects to recruit stem cells from the bone marrow, thereby promoting cartilage regeneration in the knee joint. Increasing the exposed bone marrow surface area (more holes in the same area) can enhance stem cell outflow. However, when the exposed area is large, it may affect the mechanical strength of the bone at the site of the cartilage defect. The purpose of this study is to use the finite element method to analyze the effects of drilling diameter, hole spacing, and drilling depth during microfracture surgery on the stability of the bone structure at the cartilage defect site.

**Methods:**

In this study, a normal knee joint model was selected for solid modeling, and a model of a femoral medial condyle cartilage defect was constructed. Microfracture holes with different diameters (1.0 mm, 2.0 mm, 3.0 mm), depths (10 mm, 30 mm), and spacings (1.0 mm, 2.0 mm, 3.0 mm) were created in the femoral medial condyle cartilage defect model. Using Ansys software, the knee joint’s loading conditions in the standing position were simulated, and the structural stability of the model was analyzed. The holes in areas of stress concentration were selected for more detailed mechanical analysis.

**Results:**

The Von Mises stresses for all the drilling parameters did not exceed the yield strength of the bone. Changes in the drilling parameters did not affect the bone structure around the holes. When smaller diameter drilling tools with closer spacing were used, the average maximum Von Mises stress and the average Von Mises stress on the holes were the lowest.

**Conclusion:**

Although the optimal combination of drilling parameters was not determined, this study provides a mechanical reference for the effects of drilling parameters on bone quality. It demonstrates that using smaller diameter drilling tools with closer spacing in areas of the same defect size results in a greater number of holes, with a lesser impact on bone stability. This study provides a mechanical reference for microfracture drilling.

## Background

Knee cartilage injuries can be categorized as acute or chronic. Acute injuries usually manifest as full-thickness cartilage lesions, whereas chronic injuries progress through various stages, eventually leading to subchondral bone proliferation and sclerosis once the cartilage is completely worn away (Lieberthal et al., 2015; Hunziker, 2002). Various clinical treatment methods are currently available, such as microfracture or drilling, autologous chondrocyte implantation, and mosaicplasty (Solheim et al., 2018; Na et al., 2019). Despite recent advances in treatment, microfracture remains the standard surgical technique for cartilage defects due to its cost-effectiveness, simplicity, and studies indicating its effectiveness in larger cartilage defects (Mithoefer et al., 2009). Microfracture or drilling techniques stimulate subchondral bone marrow outflow, promoting cartilage repair by recruiting bone marrow stem cells to the lesion site through the use of awls or Kirschner wires of a certain diameter (Mithoefer et al., 2009).

Numerous studies have shown that the number of mesenchymal stem cells (MSCs) migrating to the cartilage defect after microfracture influences cartilage regeneration outcomes (Na et al., 2019; Mithoefer et al., 2009). The number of MSCs in the lesion area and the therapeutic results vary depending on the size, number, and depth of the holes (Powers et al., 2021). Research has indicated that the spacing between microfracture holes can impact cartilage repair, with denser holes promoting the formation of type II collagen (Orth et al., 2016). Benthien and colleagues found that the depth of microfracture holes affects microcracks and bone compression around the holes, which in turn influences the connectivity of bone marrow around the holes (Benthien and Behrens, 2013). Increasing the surface area of bone marrow stimulation by adjusting the size and number of holes in the subchondral bone plate leads to a greater outflow of MSCs from the microfracture holes. In other words, a larger exposed area is conducive to the release of MSCs. However, since the knee bears the body’s weight, too many holes may compromise the structure of the subchondral bone and trabeculae, potentially hindering cartilage regeneration. Currently, most physicians perform microfracture drilling with a hole spacing of 3–4 mm and a depth of 3 mm, based on empirical practice, while the hole diameter is determined by the tools used. However, such microfracture drilling parameters do not adequately meet clinical requirements. Drilling depths of 10 mm or even 30 mm are necessary to effectively penetrate sclerotic bone, facilitate the release of mesenchymal stem cells, and alleviate intramedullary pressure (Benthien and Behrens, 2013). At present, the mechanical interactions among the three key microfracture parameters-holes diameter, spacing, and depth-have not been validated to determine their impact on post-microfracture bone structural stability. Biomechanical studies are needed to investigate the effects of holes diameter, spacing, and depth on bone structure.

To determine the mechanical stability after microfracture, we hypothesized that the distance between holes, the depth, and the hole size in microfracture drilling are associated with the structural stability of the bone in the drilled area. Using MRI-based modeling techniques, we constructed a model of the knee joint, and finite element analysis was applied to examine the mechanical effects of different drilling parameters in microfracture surgery. This allowed us to assess the structural stability of the bone in the drilled area.

## Methods

### Construction of knee joint and medial femoral cartilage defect models

In accordance with the standards for finite element analysis model construction, a healthy female volunteer without a history of knee joint diseases was recruited. The participant, aged 50, with a height of 160 cm and a weight of 60 kg, had no history of osteoporosis, lower limb fractures, or malalignment, and provided informed consent for the study. The DICOM format MRI data of the volunteer’s knee joint was imported into Mimics 21.0 software, and the right lower limb was selected for model reconstruction. In Mimics 21.0, a new mask was created to perform threshold segmentation of the knee joint, with the threshold set between 198 and 3,071 Hounsfield units (HU). The femur, tibia, and fibula were separated into individual masks using the separation mask tool. Subsequently, the femur, tibia, and fibula models were repaired, sealed, and filled using the add and subtract functions within the mask editing tool.

For soft tissues such as cartilage, menisci, and ligaments, the 3D models were manually extracted. After completing these steps, the extracted 3D models in STL format were imported into Geomagic Wrap 17.0 software for feature removal and smoothing. The models were further refined by adjusting contour lines, surface patches, and constructing grids and NURBS surface fitting, after which the solid 3D models of bone and soft tissues were saved in STEP format.

**FIGURE 1:**
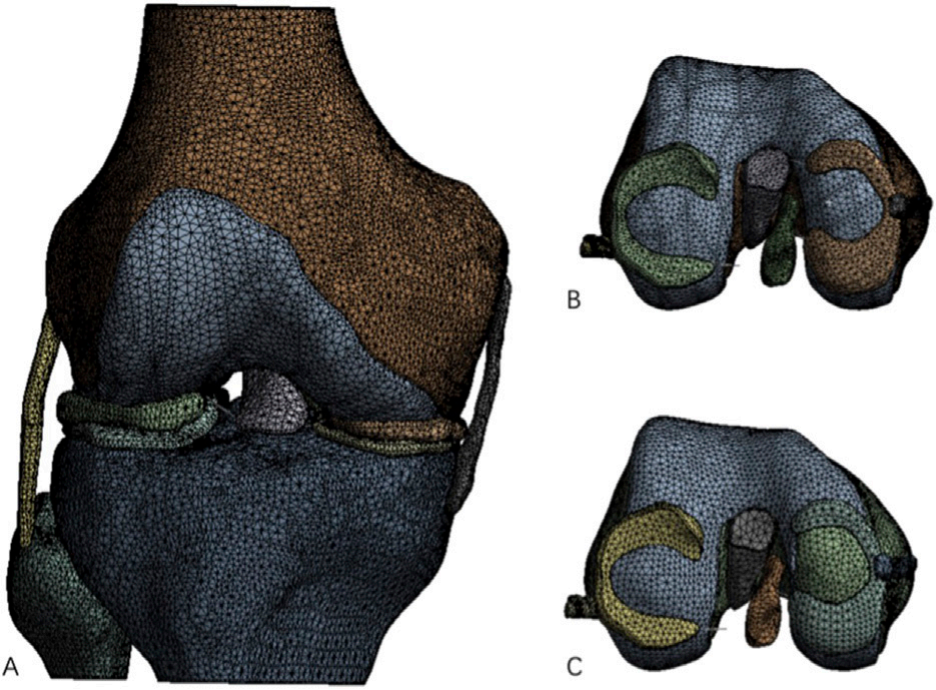
Complete knee joint model and knee joint model after construction of cartilage defect. **(A)** Processed complete knee joint model; **(B)** Intact femoral medial cartilage model; **(C)** Femoral medial cartilage defect model.

Next, the femoral cartilage solid 3D model was re-imported into Geomagic Wrap 17.0 software, where a model of a full-thickness medial condyle cartilage defect with a diameter of 20 mm was constructed (Figure 1) and saved in STEP format.

**FIGURE 2:**
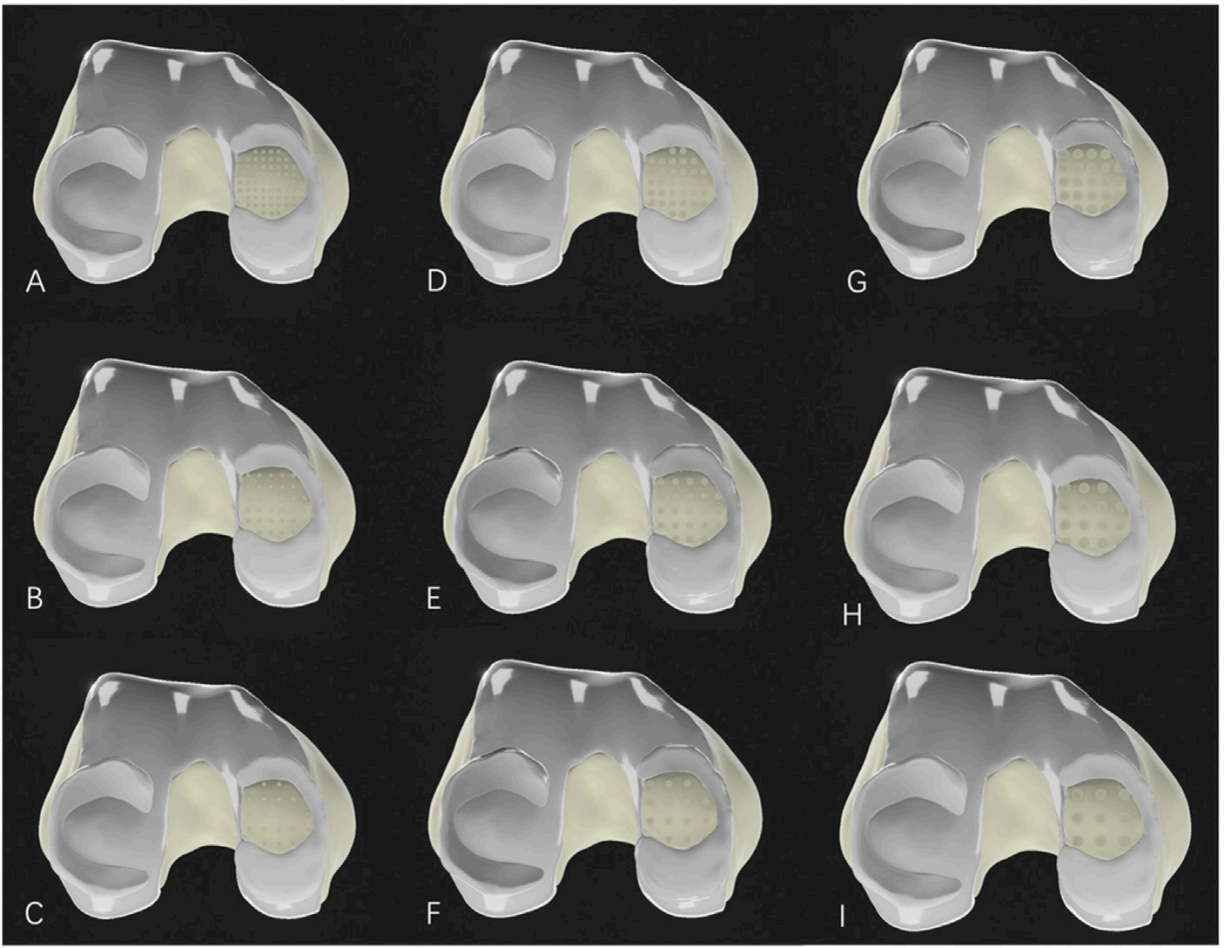
Medial femoral cartilage defect model after microfracture drilling. **(A–C)**: Microfracture models with a hole diameter of 1.0 mm; **(D–F)**: Microfracture models with a hole diameter of 2.0 mm; **(G–I)**: Microfracture models with a hole diameter of 3.0 mm. **(A, D, G)** represent drilling hole spacing of 1.0 mm; **(B, E, H)** represent drilling hole spacing of 2.0 mm; and **(C, F, I)** also represent drilling hole spacing of 3.0 mm.

The medial condyle cartilage defect model was then combined with the femur, and drilling models with different parameters were constructed in SolidWorks 2021 software (parameters listed in Table 1). These drilling models were assembled with the corresponding bone and soft tissue models for further analysis (Figure 2).

**TABLE 1:** Microfracture drilling parameters.

Drilling diameter (mm)	1.0	2.0	3.0
Drilling Spacing (mm)	1.0	2.0	3.0
Drilling depth (mm)	10	30	

### Finite element section

The lower limb cartilage defect model was imported into Ansys Workbench 2021 for meshing. The mesh type used was C3D4 tetrahedral elements, with different mesh sizes applied to various parts of the model. The tibia, fibula, medial and lateral collateral ligaments, as well as the anterior and posterior cruciate ligaments, had a mesh size of 3.0 mm, while the femur, femoral cartilage, medial and lateral tibial cartilage, and medial and lateral menisci had a mesh size of 2.0 mm. It was assumed that all models exhibited homogeneous, isotropic, and linear elastic behavior. For the material properties, the mechanical attributes of the knee joint models were derived from literature reports. Different material properties were assigned according to the stiffness of the bone, cartilage, menisci, and ligaments (Li et al., 2018; Ding et al., 2021) (Table 2), with all models defined as linear elastic, homogeneous, and isotropic materials. The connections between the menisci and the tibial plateau were replaced by one-dimensional linear spring elements (Koh et al., 2018). One spring element connected the anterior and posterior horns of the menisci to the tibia, with a spring stiffness set to 2000 N/mm.

**TABLE 2:** Material properties for all models in this study.

Material	Young’s modulus (Mpa)	Poisson’s ratio
Bone (Li et al., 2018)	7,300	0.3
Cartilage (Ding et al., 2021)	5	0.46
Meniscus (Ding et al., 2021)	59	0.49
Ligament (Ding et al., 2021)	215	0.46

In the assembled model, there were a total of 20 contact interactions: 8 were defined as “frictionless,” and 14 as “bonded.” The contact area between the femoral medial condyle cartilage and the medial meniscus was defined as Surface 1, the contact area between the femoral lateral condyle cartilage and the lateral meniscus as Surface 2, the contact area between the femoral medial condyle cartilage and the medial tibial cartilage as Surface 3, and the contact area between the femoral lateral condyle cartilage and the lateral tibial cartilage as Surface 4. The contact area between the medial tibial cartilage and the medial meniscus was defined as Surface 5, and between the lateral tibial cartilage and the lateral meniscus as Surface 6. The contact area between the bone tissue at the medial femoral cartilage defect and the medial tibial cartilage was defined as Surface 7, while the contact area between the bone tissue at the medial femoral cartilage defect and the medial meniscus was defined as Surface 8. These contact relationships were set as “hard contact between surfaces,” with the contact surfaces defined as nonlinear, “frictionless finite sliding,” to simulate the limited sliding motion of the knee joint. The remaining bone-to-ligament and bone-to-cartilage contact relationships were set as “bonded.”

To simulate the actual loading conditions on the knee joint while standing, an axial compressive load was applied to the entire lower limb. The load was applied along the force line of each model, directed downward from the proximal cross-section of the femur, with a magnitude of 600 N. The distal ends of the tibia and fibula were fixed as support constraints. During the load application, in order to maintain axial load application, the distal ends of the tibia and fibula were fully constrained in all six degrees of freedom, while only the Y-axis of the femur was fixed to prevent flexion.

## Results

### Finite element stress contour maps of knee joint models with different microfracture drilling parameters

In the normal knee joint, the highest von Mises stress in the medial tibial cartilage occurred in the anteromedial region, while the highest von Mises stress in the lateral tibial cartilage was distributed in the central region. The stress distribution in the femoral cartilage matched that of the corresponding tibial cartilage, with higher stress on the medial side compared to the lateral side. In the cartilage defect model, the exposed medial femoral condyle was in direct contact with the medial tibial cartilage, causing minor changes in the stress distribution on the lateral femoral cartilage. The average von Mises stress in the contact area of the lateral femoral cartilage decreased from 0.0427 MPa to 0.0339 MPa. The highest von Mises stress in the medial femoral condyle at the defect site was 1.6563 MPa, with an average von Mises stress of 0.6382 MPa. After microfracture drilling, the average maximum von Mises stress increased to 4.6443 MPa, with an average von Mises stress of 1.2061 MPa.

**FIGURE 3:**
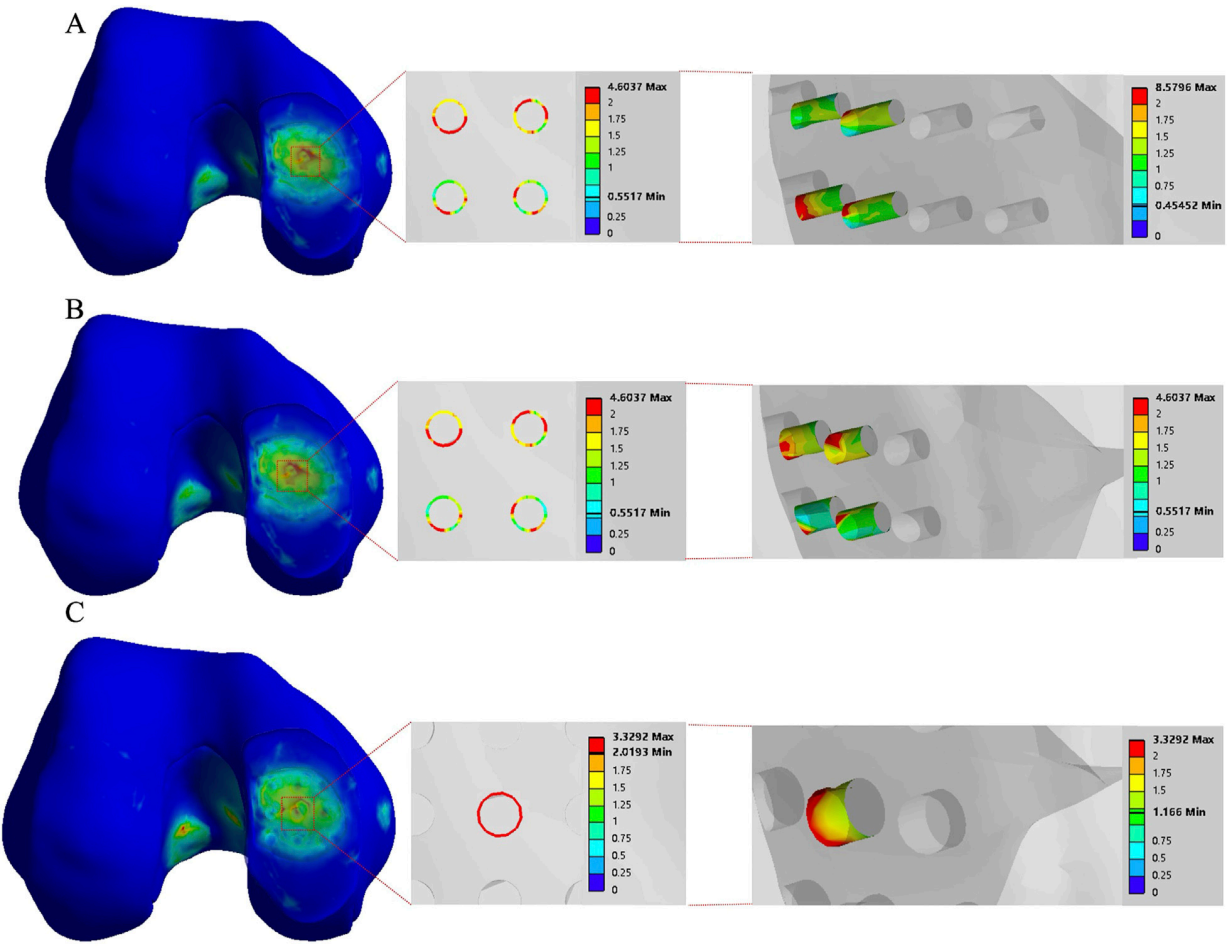
The finite element results of the conventional group with a hole spacing of 3.0 mm and a hole depth of 3.0 mm. **(A–C)** correspond to hole diameters of 1.0 mm, 2.0 mm, and 3.0 mm, respectively.

**FIGURE 4:**
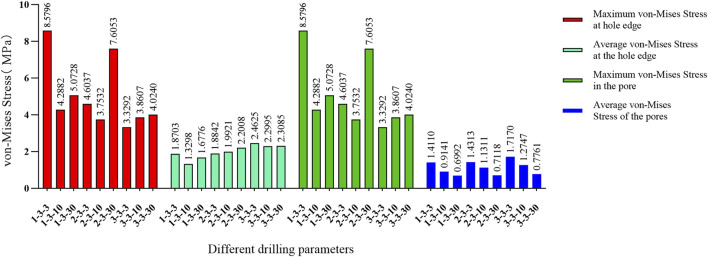
Comparison of von Mises forces under different hole diameters: hole spacing of 3.0 mm and depth of 3.0 mm *versus* hole spacing of 3.0 mm with depths of 10 mm and 30 mm, respectively.

**FIGURE 5:**
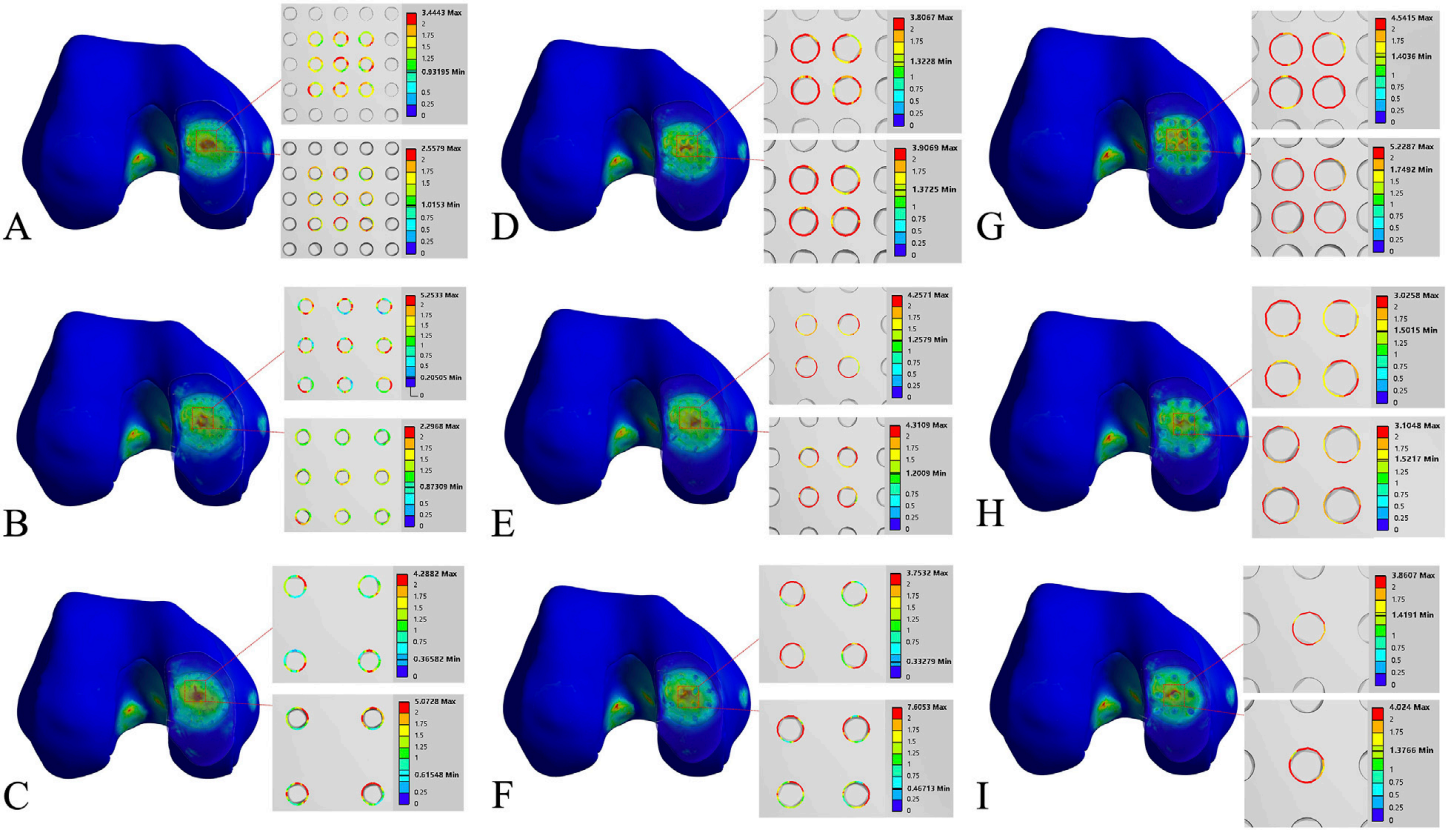
Finite element contour maps for different drilling parameters. **(A–C)**: 3D finite element contour maps of the knee joint after microfracture with a hole diameter of 1.0 mm, showing 10 mm hole depth (top) and 30 mm hole depth (bottom); **(D–F)**: 3D finite element contour maps of the knee joint after microfracture with a hole diameter of 2.0 mm, displayed in the same format; **(G–I)**: 3D finite element contour maps of the knee joint after microfracture with a hole diameter of 3.0 mm.

The stress concentration region within the medial compartment of the knee joint was selected for further analysis, focusing on the differences in the number of drilling holes within the stress region caused by variations in hole diameter and spacing. Depending on the drilling parameters, the stress concentration region contained 9 holes, 4 holes, or 1 hole. Due to the sharp edges of the holes, stress concentration is more likely to occur, with the maximum stress location identified for each model. The finite element results of the conventional microfracture model group showed no significant difference compared to the experimental group. However, under the same hole diameter and spacing conditions, the average von Mises stress in the channels exhibited a decreasing trend with increasing hole depth (Figures 3, 4). In the experimental group, the maximum von Mises stress at the drilled holes in the stress concentration region was 7.6053 MPa, with an average maximum von Mises stress of 4.1299 MPa. Different drilling depths resulted in variations in the maximum von Mises stress at the same drilling location (Figure 5).

**FIGURE 6:**
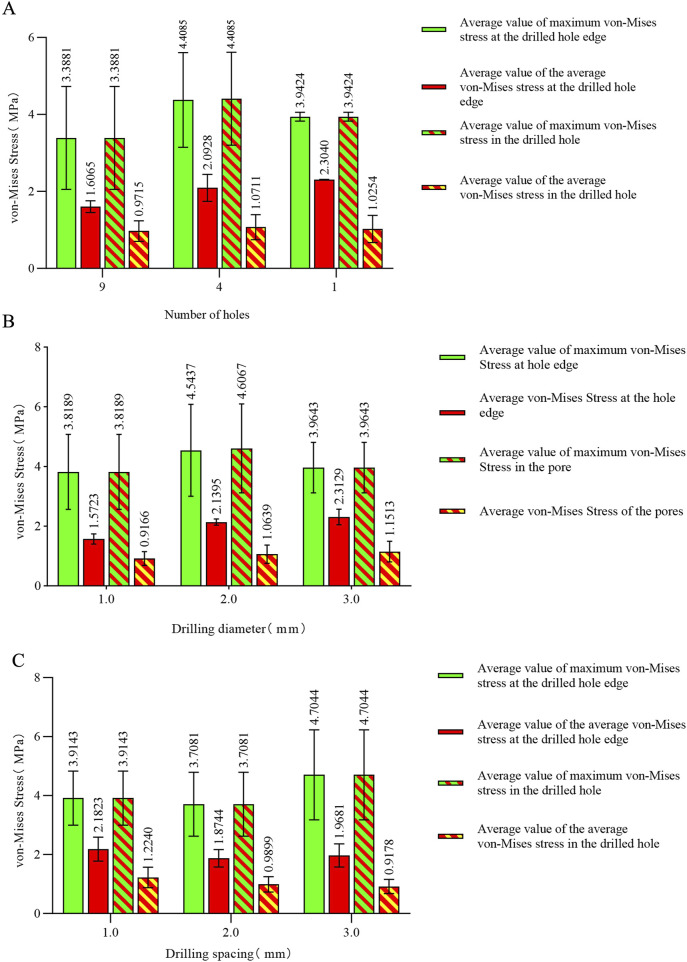
**(A)** Von Mises stress data for different numbers of holes in the stress concentration area; **(B)** von Mises stress data for different hole diameters in the stress concentration area; **(C)** von Mises stress data for different hole spacings in the stress concentration area.

The maximum von Mises stress for 9 holes was 5.2533 MPa, with an average maximum von Mises stress of 3.3881 MPa. For 4 holes, the maximum von Mises stress was 7.6053 MPa, with an average maximum von Mises stress of 4.3770 MPa. For 1 hole, the maximum von Mises stress was 4.024 MPa, with an average maximum von Mises stress of 3.9424 MPa. The maximum von Mises stress for hole diameters of 1.0 mm, 2.0 mm, and 3.0 mm was 5.2533 MPa, 7.6053 MPa, and 4.024 MPa, respectively, with corresponding average maximum von Mises stresses of 3.8189 MPa, 4.6067 MPa, and 3.9643 MPa. The maximum von Mises stress for hole spacings of 1.0 mm, 2.0 mm, and 3.0 mm was 5.2287 MPa, 5.2533 MPa, and 7.6053 MPa, respectively, with corresponding average maximum von Mises stresses of 3.9143 MPa, 3.7081 MPa, and 4.7674 MPa (Table 3–6; Figure 6).

**TABLE 3:** Von mises stress data of stress concentration area with different drilling parameters.

Microfracture drilling parameters (mm)	Number of drill holes in the stress concentration area	Maximum von mises stress at the edge of the hole in the stress area (MPa)	Average von mises stress at the edge of the hole in the stress area (MPa)	Maximum von mises stress in the pores of the stress area (MPa)	Average von mises stress in the pores of the stress area (MPa)
1,1,10	9	3.4443	1.686	3.4443	1.2975
1,1,30	9	2.5579	1.7288	2.5579	0.83991
1,2,10	9	5.2533	1.6232	5.2533	1.0648
1,2,30	9	2.2968	1.3881	2.2968	0.68387
1,3,10	4	4.2882	1.3298	4.2882	0.91412
1,3,30	4	5.0728	1.6776	5.0728	0.69922
2,1,10	4	3.8067	2.2081	3.8067	1.5168
2,1,30	4	3.9069	2.269	3.9069	0.9123
2,2,10	4	4.2571	2.0626	4.2571	1.302
2,2,30	4	4.3109	2.1045	4.3109	0.80924
2,3,10	4	3.7532	1.9921	3.7532	1.1311
2,3,30	4	7.6053	2.2008	7.6053	0.71176
3,1,10	4	4.5415	2.5162	4.5415	1.7103
3,1,30	4	5.2287	2.6854	5.2287	1.0673
3,2,10	4	3.0258	1.9985	3.0258	1.2715
3,2,30	4	3.1048	2.0695	3.1048	0.8078
3,3,10	1	3.8607	2.2995	3.8607	1.2747
3,3,30	1	4.024	2.3085	4.024	0.77605

**TABLE 4:** Von mises stress data in the stress concentration area for different numbers of drill holes (1).

Number of holes in the stress concentration area	Maximum von mises stress at the edge of the hole in the stress area (MPa)	Average von mises stress at the edge of the hole in the stress area (MPa)	Maximum von mises stress in the pores of the stress area (MPa)	Average von mises stress in the pores of the stress area (MPa)
9	3.3881	1.6065	3.3881	0.9715
4	4.4085	2.0928	4.4085	1.0711
1	3.9424	2.304	3.9424	1.0254

**TABLE 5:** Von mises stress data in the stress concentration area for different numbers of drill holes (2).

Drilling diameter (mm)	Maximum von mises stress at the edge of the hole in the stress area (MPa)	Average von mises stress at the edge of the hole in the stress area (MPa)	Maximum von mises stress in the pores of the stress area (MPa)	Average von mises stress in the pores of the stress area (MPa)
1.0	3.8189	1.5723	3.8189	0.9166
2.0	4.6067	2.1395	4.6067	1.0639
3.0	3.9643	2.3129	3.9643	1.1513

**TABLE 6:** Von mises stress data in the stress concentration area for different numbers of drill holes (3).

Drilling spacing (mm)	Maximum von mises stress at the edge of the hole in the stress area (MPa)	Average von mises stress at the edge of the hole in the stress area (MPa)	Maximum von mises stress in the pores of the stress area (MPa)	Average von mises stress in the pores of the stress area (MPa)
1.0	3.9143	2.1823	3.9143	1.2240
2.0	3.7081	1.8744	3.7081	0.9899
3.0	4.7674	1.9681	4.7674	0.9178

**FIGURE 7:**
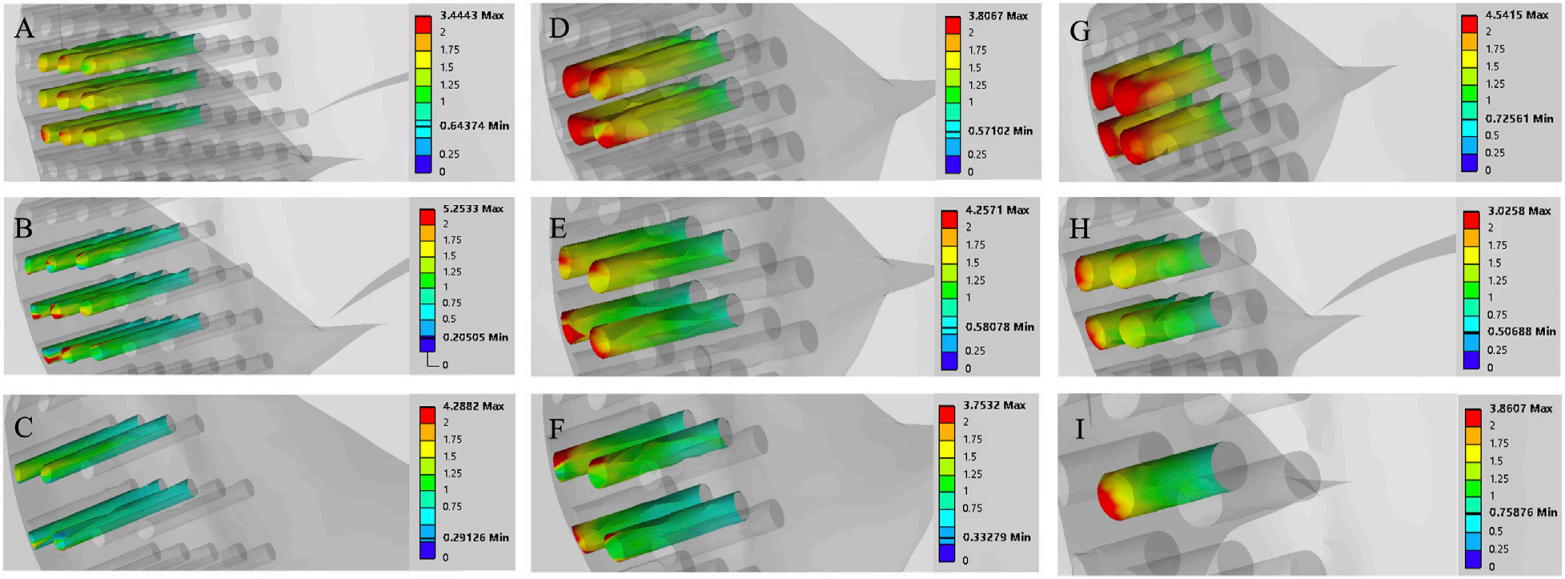
Finite element contour maps for different drilling parameters with a hole depth of 10 mm. **(A–C)**: 3D finite element contour maps of the knee joint after microfracture with a hole diameter of 1.0 mm; **(D–F)**: 3D finite element contour maps of the knee joint after microfracture with a hole diameter of 2.0 mm; **(G–I)**: 3D finite element contour maps of the knee joint after microfracture with a hole diameter of 3.0 mm.

**FIGURE 8:**
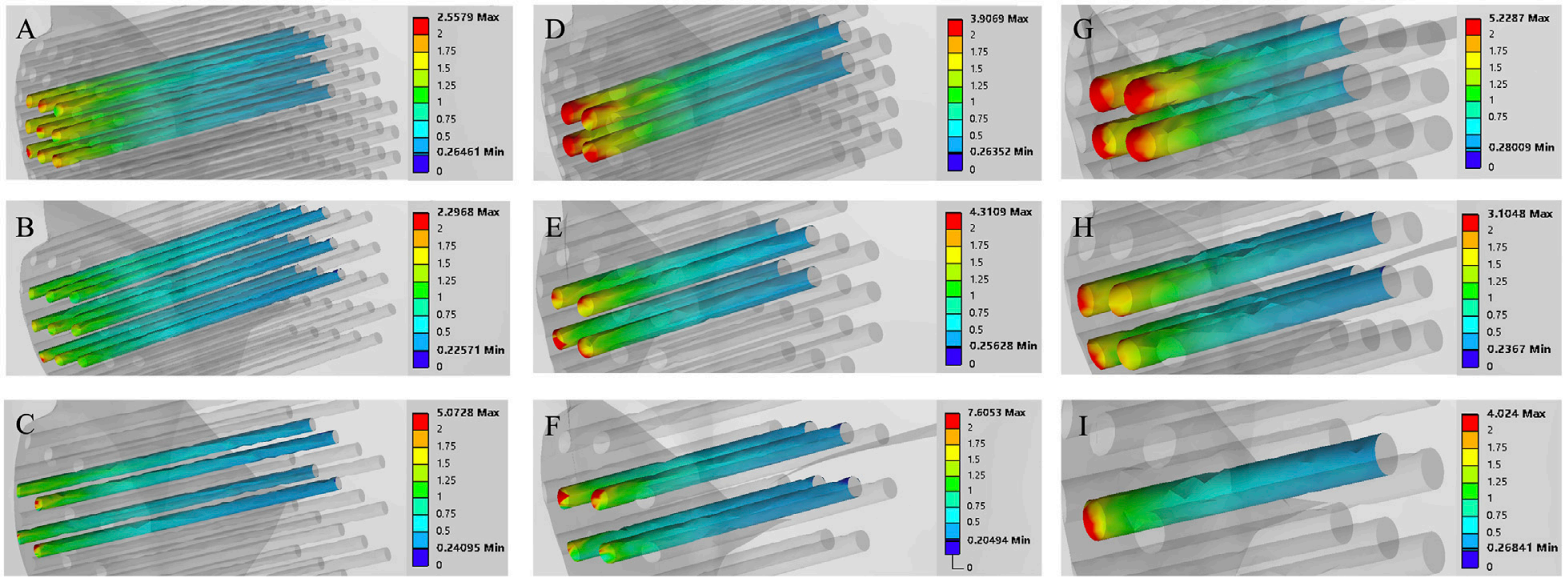
Finite element contour maps for different drilling parameters with a hole depth of 30 mm. **(A–C)**: 3D finite element contour maps of the knee joint after microfracture with a hole diameter of 1.0 mm; **(D–F)**: 3D finite element contour maps of the knee joint after microfracture with a hole diameter of 2.0 mm; **(G–I)**: 3D finite element contour maps of the knee joint after microfracture with a hole diameter of 3.0 mm.

**FIGURE 9:**
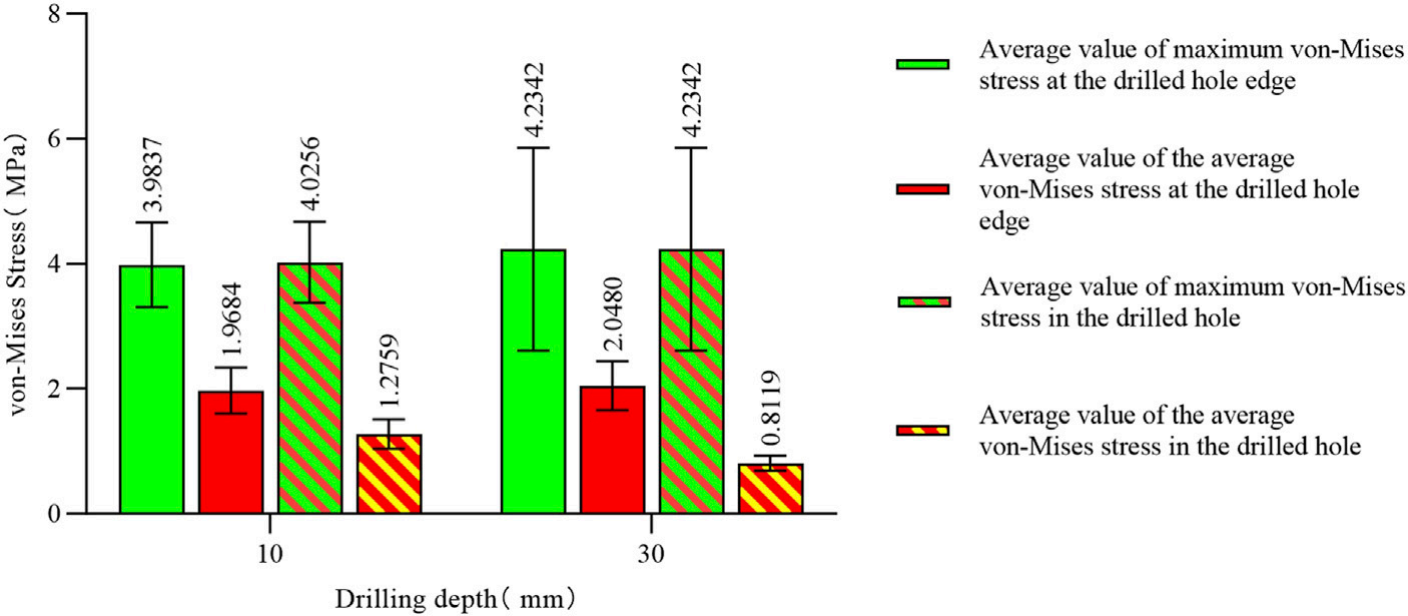
Von Mises stress data for different hole depths in the stress concentration area.

The maximum von Mises stress for a hole depth of 10 mm and a hole depth of 30 mm was the same at the hole edge, both occurring at the contact location. Changing the depth did not affect the maximum von Mises stress value but did influence the average stress in the hole channel (Figures 7, 8). The average von Mises stress for a hole depth of 10 mm was 1.2759 MPa, while for a hole depth of 30 mm, it was 0.8583 MPa (Table 7; Figure 9).

**TABLE 7:** Von mises stress data in the stress concentration area for different numbers of drill holes (4).

Drilling depth (mm)	Maximum von mises stress at the edge of the hole in the stress area (MPa)	Average von mises stress at the edge of the hole in the stress area (MPa)	Maximum von mises stress in the pores of the stress area (MPa)	Average von mises stress in the pores of the stress area (MPa)
10	4.0256	1.9684	4.0256	1.2759
30	4.2342	2.0480	4.2342	0.8119

## Discussion

Microfracture drilling is a minimally invasive and simple technique that promotes the outflow of bone marrow stem cells to the site of cartilage defects, inducing cartilage repair. Due to its advantages of fewer complications and ease of operation, it has long been the standard treatment for cartilage injuries (Peng et al., 2023). In a follow-up study of patients who underwent microfracture for 5 years, 83% reported pain relief (Kraeutler et al., 2018). Another study reported significant improvements in various knee evaluation scores following microfracture. In terms of activity, 86% of patients who underwent microfracture for traumatic osteochondral lesions were able to resume pre-injury levels of physical activity (Steadman et al., 2003).

Although it has advantages, biomechanical studies on microfracture techniques are limited. Current clinical practice typically uses microfracture holes with a diameter of 3 mm, spacing of 3–4 mm, and depth of 3–4 mm, aiming for fat droplets to appear as an endpoint (Gobbi et al., 2005; Hoemann et al., 2013). Some researchers suggest that drilling depths exceeding 10 mm may be more effective for marrow stem cell release and trabecular bone repair (Benthien and Behrens, 2013). However, there is no evidence to confirm whether microfracture drilling causes mechanical structural changes. Chen et al. proposed that drilling in areas of cartilage defects could lead to alterations in the subchondral bone and trabecular structures, potentially modifying the biomechanics of the knee joint and ultimately affecting cartilage repair. Nevertheless, this hypothesis lacks mechanical evidence (Chen et al., 2011). Some researchers have observed in animal studies that microfracture surgery may cause subchondral bone damage and increase the risk of bone cyst formation (Gao et al., 2017). However, we have not encountered any cases of bone cysts resulting from microfracture surgery in clinical practice. The appearance of bone cysts after microfracture in animal models may be attributed to the smaller skeletal size of animals. When drilling with 0.55 mm or 1.2 mm Kirschner wires, it already constitutes a significant bone defect for small animals, potentially leading to bone cyst formation and poor cartilage repair outcomes. If this is proportionally scaled to the human femoral condyle, it would also represent a substantial bone defect, necessitating autologous cartilage transplantation in such cases. Additionally, some researchers have found in animal studies that simple debridement alone can achieve cartilage repair effects similar to those of microfracture surgery (Sumii et al., 2023). Clinically, we have observed that microfracture not only facilitates cartilage repair but also alleviates subchondral bone sclerosis by releasing intramedullary pressure, thereby further relieving knee joint pain.

Microfracture is a surgical technique that creates channels in cartilage defects to allow bone marrow outflow, facilitating the aggregation of a large number of cells that promote cartilage regeneration (Kwon et al., 2019; Cooper and Rainbow, 2022). It has been hypothesized that creating larger or more channels for cellular migration could improve cartilage repair, as more mesenchymal stem cells from the bone marrow would be recruited to the lesion area. This concept has been supported by recent studies (Goh et al., 2023; Lin et al., 2017). Biomechanical studies have investigated the relationship between bone stability and microfracture holes. In this study, we used the parameters of microfracture holes as the basis for finite element analysis, grouping the variables of hole diameter, spacing, and depth.

In this study, the von Mises stress varied with changes in the parameters of the holes, but it remained well below the yield stress of bone (135 MPa). Our findings align with those of Yin et al., concluding that while drilling parameters can influence the von Mises stress, they are insufficient to induce changes in the mechanical structure of bone (Yin et al., 2020). However, there are significant differences between our finite element results and theirs, which may be attributed to differences in the models used. Yin et al. constructed a simplified knee joint stress model that included only the subchondral bone and cancellous bone, resulting in a more idealized representation compared to actual human bone (Yin et al., 2020). In contrast, we developed a complete knee joint model incorporating ligaments, menisci, and cartilage. While their applied force accounted for the effects of cartilage and menisci, we believe that constructing a full knee joint model in finite element analysis provides a more accurate simulation of real-world loading conditions. This is because these additional structures contribute to stress distribution in the mechanical environment. Furthermore, we consider bone to be a complex mechanical structure comprising cortical bone and cancellous bone. The cancellous bone contains an irregular trabecular structure and bone marrow, all of which play a role in stress distribution during loading. Therefore, we believe that analyzing bone stress through a comprehensive model with holistic material property assignments may yield more accurate and clinically relevant results. Analysis of the results revealed that the maximum von Mises stress consistently occurred at the edges of the holes, indicating that sharp edges at the hole boundaries can lead to stress concentration. The average von Mises stress within the hole decreased with increasing hole length, suggesting that longer holes help to distribute stress more effectively. Notably, statistical analysis of the maximum von Mises stress and the average von Mises stress for each parameter showed that a hole diameter of 1.0 mm, a spacing of 2.0 mm, and a depth of 30 mm resulted in the lowest values for both the maximum von Mises stress and the average von Mises stress. We also observed that the maximum von Mises stress for a 2.0 mm hole diameter was higher than that for 1.0 mm and 3.0 mm. This outcome could be explained by the fact that smaller diameter holes (1.0 mm) result in a smaller load-bearing area around the hole. As a result, under applied external forces, the force per unit area is reduced, leading to weaker stress concentration at the hole edges. Additionally, the surrounding bone maintains stronger continuity, distributing the stress more evenly. Typically, stress concentration at the hole edge initially increases with larger diameters but may subsequently decrease as larger holes provide a greater area to disperse the stress (Khechai et al., 2014; Bakhshi and Taheri-Behrooz, 2019; Masrol and Siswanto, 2014). The maximum von Mises stress observed for four holes was higher than that for nine holes and one hole, potentially due to the interaction of stress fields around the holes. With four holes, the distribution may lead to larger overlapping stress field regions, intensifying the interactions and resulting in higher von Mises stress. For a single hole, there is no interaction with other stress fields, resulting in a single stress concentration zone and relatively lower von Mises stress. For nine holes, the denser distribution might cause more stress field overlap, but the stress concentration around each hole is more evenly distributed, leading to a lower overall von Mises stress (Driscoll, 2014; Zhou et al., 2014). Smaller hole diameters are less likely to cause compression of the surrounding bone. With closer spacing between holes, more holes can be created per unit area, facilitating the release of more mesenchymal stem cells from the bone marrow. Drilling depth should be as deep as possible, as depths of 3–4 mm may not penetrate sclerotic bone in patients with subchondral bone sclerosis. It can be concluded that if the bone structure is adequately protected during microfracture, hole diameter, spacing, and depth are not risk factors within the biomechanical load range. In practical surgical applications, ensuring the strength and operability of the drilling tools, it is recommended to use smaller-diameter drilling tools, denser hole spacing, and the deepest feasible drilling depth. Such parameters not only preserve the mechanical integrity of the drilled region but also enhance the effectiveness of mesenchymal stem cell release from the bone marrow.

The limitations of this study include the lack of validation through dynamic finite element analysis. During walking, the magnitude and angle of the forces exerted on the knee vary across different phases of the gait cycle. In clinical practice, approximately 20 holes are drilled in a 20 mm cartilage defect. However, in this experiment, finite element analysis was only performed on the holes located in the directly loaded region, as the von Mises stresses on the non-contact regions were minimal. The influence of hole arrangement on von Mises stress was not discussed in detail. This study specifically analyzed the biomechanics of a single knee joint in a standing position, chosen as a more extreme and reliable condition.

The aim of this study was to analyze the relationship between bone structural stability and the parameters of microfracture holes. The results indicate that within the parameter ranges used in this study, the hole parameters do not affect structural stability. Based on this finding, using drilling tools with the smallest possible diameter and adopting a drilling approach with small spacing and deeper penetration during microfracture surgery may facilitate the release of more bone marrow-derived mesenchymal stem cells, potentially improving cartilage regeneration. However, it remains unclear whether this would yield superior clinical outcomes. We hypothesize that smaller hole diameters result in less damage to the bone surrounding the microfracture sites, and greater hole depths facilitate a more efficient release of MSCs. Further studies are required to validate the actual clinical outcomes.

## Conclusion

The limitation of this study is the absence of dynamic finite element analysis for the knee joint, which would provide a more realistic simulation of the mechanical conditions during weight-bearing activities of the knee. However, we constructed a weight-bearing knee joint model in the standing position to simulate the maximum load experienced in this posture. The results demonstrated that various microfracture drilling parameters had no effect on the mechanical structure of the bone. It is equally important to analyze the mechanical effects of microfracture drilling during gait using different lower limb models. This can be achieved by altering bone geometry to create customized lower limb models, allowing finite element analysis of changes in the mechanical conditions at the microfracture sites under varying joint angles.

## Data Availability

The original contributions presented in the study are included in the article/supplementary material, further inquiries can be directed to the corresponding author.
